# Recommendations for promoting affirming healthcare for gender and sexual minorities with intersecting marginalized identities

**DOI:** 10.1186/s12913-025-12708-7

**Published:** 2025-04-23

**Authors:** Rajinder Sonia Singh, Kim Zamarin, Kristen L. Eckstrand, Marisa Sklar, Robert Sturm, Cathleen Willging

**Affiliations:** 1 Center for Mental Healthcare and Outcomes Research, Central Arkansas Veterans Healthcare System, Little Rock, AR USA; 2 Department of Psychiatry, University of Arkansas for Medical Sciences, Little Rock, AR USA; 3 Behavioral Health Research Center of the Southwest, Pacific Institute for Research and Evaluation, Albuquerque, NM USA; 4 Department of Psychiatry, University of Pittsburgh School of Medicine, Pittsburgh, PA USA; 5 Department of Psychiatry, University of California San Diego, San Diego, CA USA; 6 UC San Diego ACTRI Dissemination and Implementation Science Center, 9500 Gilman Drive, La Jolla, CA 92093 USA; 7 Child and Adolescent Services Research Center, 3665 Kearny Villa Rd., Suite 200N, San Diego, CA 92123 USA

**Keywords:** Gender minority, Intersectionality, Health equity, Primary care, Sexual minority

## Abstract

**Background:**

Many existing implementation frameworks neglect inequity. Theories of intersectionality can help implementation researchers understand the multiplicative burden of certain inequities experienced by people with intersecting marginalized identities. The current project provides an example of engaging primary care providers, staff, and patients in prioritizing recommendations to improve services for lesbian, gay, bisexual, transgender, queer, and other gender and sexual minoritized (LGBTQ+) people from diverse racial, ethnic, cultural, and economic backgrounds.

**Methods:**

We used the Nominal Group Technique (NGT) to guide two one-time sessions with providers (*n* = 6) and staff (*n* = 8) affiliated with four primary care clinics in the United States. These participants brainstormed responses to a single focal question designed to elicit ideas for improving services for LGBTQ+ people with intersecting marginalized identities. Participants then discussed and ranked the ideas generated and considered specific strategies for ranked ideas. Finally, we conducted two focus groups with LGBTQ+ primary care patients (*n* = 7, *n* = 4) to obtain their insights into the recommendations for improving services.

**Results:**

The highest-ranked idea by providers was to mandate ongoing high-quality professional development for primary care personnel. The highest-ranked ideas by staff were to offer safe spaces characterized by an ambient atmosphere with trained personnel and LGBTQ+ visuals and to increase availability and funding for transgender providers and services delivered by transgender people and others skilled in caring for this community. Patients affirmed the recommendations from the NGT, while emphasizing inclusive representation in primary care spaces and for providers and staff to critically reflect of their own backgrounds.

**Conclusions:**

Providers, staff, and patients highlighted the importance of continuing education and training to offer affirming, safe, and equitable care for LGBTQ+ people with intersecting marginalized identities. These implementation suggestions may be helpful for primary care clinics in developing inclusive and equitable medical environments. Further, the NGT, followed by a review of findings by impacted patients, may be useful when considering equitable implementation focused on meeting the needs of people with intersecting marginalized identities.

## Contributions to the literature

Lesbian, gay, bisexual, transgender, queer, and other gender and sexual minoritized (LGBTQ+) people from diverse racial, ethnic, cultural, and economic backgrounds experience barriers to equitable primary care due to systemic discrimination based on their intersecting marginalized identities. We can improve care by educating providers and staff about their needs, creating supportive clinical environments, and promoting critical reflection and inclusive caregiving. Greater representation of LGBTQ+ people, particularly transgender people, in the primary care workforce is important. We used community-engaged methods to center the experience of LGBTQ+ people with intersecting marginalized identities and prioritize recommendations for increasing patient equity in primary care.

## Background

Disparities in health and healthcare result from inequities of power and privilege and can engender inequitable treatment [1, 2]. In implementation science, neglecting the power relations that sustain health disparities may worsen implementation outcomes and health inequities [3]. Health equity is an ideal state where people receive needed care to optimize their well-being. Disparities in implementation occur when intervention (e.g., treatment) delivery significantly undermines access, quality, or outcomes for certain populations compared to others [3, 4]. Implementation science must address factors contributing to health inequities [5]. Determinants of equitable implementation are often structural (e.g., racism) [6, 7]. Disparities in implementation are often shaped by structural factors and occur when intervention delivery significantly undermines access, quality, or outcomes for certain populations compared to others.

There are also disparities in interventions; if interventions are not implemented with equity in mind, they may increase disparities. For example, if two sites are selected to implement an intervention, with one being more resourced and one being less resourced, the one that is less resourced may be deemed an “implementation failure.” That implementation failure may no longer receive the intervention or implementation support, thus rewarding the more resourced environment and punishing the less resourced environment that needs more implementation support. The result of this inequitable implementation would worsen access to the intervention and, therefore, maintain or increase disparities. There are calls for equity in implementation science [3]. One way to answer this call is to consider factors related to health equity in implementation [8]. 

Intersectionality posits that individuals’ experiences and identities overlap [9]. Intersectionality theory was developed by Black feminist and critical race scholars [8–10], emphasizing how multiple aspects of power and difference shape an individual’s positionality [9]. This theory provides the lens on how intersecting systems of oppression impact the experience of individual’s overlapping identities. Intersectionality challenges the idea of universal, fixed categories of identity (e.g., separating different identities such as Black and queer). Instead, it highlights how marginalization based on race, gender, and sexuality, as well as several other identities, interact with one another [11, 12]. These overlapping experiences of marginalization are viewed as multiplicative and not just additive [11–14]. Intersectionality provides a framework for how intersecting systems of oppression may lead to the development of health disparities for patients based on the compounding effects of marginalization [11–14]. Such patients may include lesbian, gay, bisexual, transgender, queer, and other gender and sexual minoritized + (LGBTQ+) people who experience poorer health compared to their heterosexual and cisgender counterparts [15–17]. 

LGBTQ+ people with intersecting marginalized identities are disproportionately impacted by health conditions often identified in primary care [18–20]. However, LGBTQ+ people are less likely to access preventative services and treatment [6]. These health disparities and reduced access are caused by societal discrimination, including in healthcare settings [6, 21]. In addition to health disparities, LGBTQ+ people with intersecting marginalized identities experience healthcare disparities when discrimination occurs in healthcare settings [22, 23]. LGBTQ+ people with intersecting marginalized identities may delay/avoid healthcare, experience health complications, and/or develop a mistrust of healthcare institutions as a result of discrimination in healthcare settings [24]. 

The goal of primary care is to follow a person-centered approach inclusive of all people [25]. Person-centered approaches may help facilitate inclusive and affirming care for all populations. As the frontline of healthcare, primary care professionals are suited to address disparities as they are trained in prevention, screening, and treatment services [26]. However, providers often report discomfort in providing LGBTQ+ care or lack resources to meet the needs of LGBTQ+ patients [27–29]. Additionally, providers often lack training in cultural humility in treating LGBTQ+ patients in general, as well as those with marginalized intersecting identities [30]. LGBTQ+ patients with marginalized intersecting identities also identify barriers to accessing primary care, such as encountering stigma and discrimination from their healthcare providers [31–33]. 

In general, the field of implementation science is moving towards considering health equity. However, health equity efforts within implementation science are often still specific to a single population. This study considers an intersectional approach to improving primary care services for LGBTQ+ patients with intersecting marginalized identities. Audre Lorde stated, “There is no such thing as a single-issue struggle because we do not live single-issue lives” [34]. This study also presents methods to consider how intersecting marginalization may affect patients and multiple solutions to a multifaceted problem.

We considered solutions to barriers from the people with lived experience (LGBTQ+ people seeking healthcare) and providers and staff working in primary care settings. We used the Nominal Group Technique (NGT) to brainstorm ideas for improving primary care services for LGBTQ+ patients from diverse racial, ethnic, cultural and economic backgrounds. We examine the extent to which LGBTQ+ patients in focus groups agreed with the ideas prioritized by providers and staff and discuss the recommendations offered by participants.

## Methods

This paper is part of a larger mixed-method project examining LGBTQ+ affirming care in primary care in three federally qualified healthcare centers (FQHCs) and one academic setting [27]. An FQHC is a safety-net system – that is a community-based healthcare organization funded by the U.S. Health Services and Resources Administration to deliver primary care in underserved areas. Recruitment focused on clinics serving low-income and racially/ethnically diverse patients. In the larger study, a systematic review of practice guidelines/recommendations and several focus groups, interviews, and NGT groups were conducted to enhance primary care services for LGBTQ+ patients in general. In this study, we report only on a subset of these data specific to LGBTQ+ patients with marginalized intersecting identities.

The Institutional Review Board at the senior author’s institution approved this study. All research activities were conducted in accordance with the Declaration of Helsinki. Researchers met with administrators at each site. Administrators met with personnel to determine if the clinic would participate. We recruited providers, staff and LGBTQ+ patients from these sites. All participants completed an informed consent process prior to enrolling in the study. All participants received $50 and travel reimbursement. A Scientific Advisory Board (SAB) of LGBTQ+ patients, providers, healthcare advocates, and researchers guided this work.

### Providers and staff

We conducted two one-time, two-hour, in-person NGT sessions in October 2019. Participants were recruited via flyers and email invitations and included providers (*n* = 6) and staff (*n* = 8) from the study sites (Table 1). Eligible providers/staff must have worked at the FQHC for one or more year(s) for an average of at least 20 h per week to ensure familiarity with clinical procedures and context-specific healthcare needs. Moderators were public health researchers who asked participants (based on SAB input), “What are your recommendations to improve services for LGBTQ+ clients of diverse racial, ethnic, cultural and economic backgrounds?”

**Table 1:** Provider and staff demographics

Demographic Domain	Totals, *n*
Total Participants	14
Professional role	
Providers	6
Administrators	2
Frontline Staff	6
Gender Identity	
Female-identified	12
Male-identified	2
Race	
African American	1
White	10
Another race not listed	3*
Ethnicity	
Hispanic/Latino	6
Not Hispanic/Latino	8
Sexual Orientation	
Bisexual	2
Lesbian/gay	2
Heterosexual	9
Queer	1
Prefer Not to Disclose	1
Average Age	46.5
Average Years Worked in Healthcare	12.6
Average Years at Current Job	4

*Another race/ethnicity not listed responses were Mexican-American, Spanish, and Black Hispanic

The NGT is a structured variation of a small-group discussion for reaching a consensus. During the NGT, a moderator asks participants to respond to a focal question and then prioritize ideas from the group [35, 36]. This approach emphasizes the collaborative sharing of ideas and prevents an individual from heavily influencing the discussion. The NGT includes four steps: (1) generating ideas, (2) recording ideas, (3) discussing ideas, and (4) voting on ideas [35]. In Step 1, participants considered the focal question and wrote responses individually. For Step 2, participants engaged in a round-robin sharing of recorded responses that were sequentially numbered. In Step 3, moderators asked all participants if they had questions about each idea expressed. In Step 4, the participants voted individually on each idea and prioritized them from “5” (highest) to “1” (lowest). They were given five sheets of paper and instructed to write down their five prioritized ideas. Next, they spread out the sheets and selected the one containing the most important idea, placing a “5” in the lower right-hand corner. They considered which idea on the remaining four sheets was least important, placing a “1” in the same corner. They used this same procedure to vote on the next important idea (“4”) and least important idea (“2”), with the idea on the last sheet assigned “3.” The moderator collected the sheets, scrambled them, recorded the priority numbers on a flip chart for each idea, and tallied numbers to identify the recommendations ranked highest numerically. Participants were then asked, “What do you think are the best ways to implement this recommendation in your clinics?”

### LGBTQ+ patients

As a triangulation strategy to enhance the credibility of the findings from NGT sessions [37], we organized two focus groups of LGBTQ+ patients (*n* = 7, *n* = 4) to reflect on the recommendations (Table 2).

**Table 2:** LGBTQ+ patient demographics

Demographic Domain	Totals, *n*
Total participants	11
Sex (Assigned at Birth)	
Female	3
Male	6
Intersex or Unsure	2
Gender Identity (Current)	
Female	2
Male	2
Trans man	1
Trans woman	4
Genderqueer or Gender Nonconforming	2
Different identity	2
Race	
American Indian	1
White	7
Another race not listed	3*
Declined	1
Ethnicity	
Hispanic/Latino	2
Not Hispanic/Latino	9
Sexual Orientation	
Bisexual	2
Heterosexual	-
Lesbian/Gay	4
Queer	4
Different orientation	4
Declined	-
Employment Status	
Employed full-time	1
Employed part-time	2
Student	1
Military	1
Disabled	4
Unemployed	2
Average Age	32
Age Range	25–46

*For another race not listed, participants responded Caucasian, Spanish descent, and Ashkenazi Jewish

To recruit, we advertised at clinic sites and worked with our SAB and local LGBTQ+ organizations. Inclusion criteria included LGBTQ+ identifying, age 18 or older, having visited a primary care clinic in the past five years, and interest in improving primary care for LGBTQ+ patients. Intersectional marginalized identity was not an inclusion requirement, given the larger study’s focus on LGBTQ+ affirming care in safety-net settings like FQHCs that serve low-income and culturally diverse populations. The first two-hour, audio-recorded and transcribed discussion occurred in an academic setting; the second in a private, community setting.

Participants were asked about their primary care experiences and their clinic’s preparation for serving LGBTQ+ patients. Key stem questions included: 1) What is it like to receive primary care in your community as an LGBTQ+ person? 3) How prepared is your primary care clinic to best serve LGBTQ patients? 4) How prepared is your primary care to care for LGBTQ+ patients of different cultural, racial, or ethnic backgrounds? 5) How prepared is the clinic to care for LGBTQ patients of different economic backgrounds? 6) Based on your experience, what are the main things primary care clinics can do to improve access and quality of care for LGBTQ patients of diverse backgrounds? Questions also inquired into how the location of clinics (e.g., rural and urban; town and city) and other axes of diversity (e.g., patient age) might affect care. We also asked about ideas from the NGT sessions for improving primary care for LGBTQ+ patients with intersection identities patients, in addition to their own recommendations.

We analyzed focus group data using inductive and deductive steps to identify themes [38]. First, we reviewed transcripts for overall content. Second, text segments were assigned codes based on questions and topics in the interview guide. Third, we used open coding to identify issues not previously considered and focused coding on which issues recurred or represented unique or unusual concerns to participants [39]. Fourth, we grouped codes with similar content into broader themes linked to segments of text. We reviewed this work with the research team and collectively resolved discrepancies.

## Results

### Provider and staff recommendations

The providers generated a total of 30 recommendations and staff a total of 25 recommendations (Appendix 1). For providers, the top three identified recommendations in order of priority ratings were:

1. **Mandate ongoing professional development for providers and staff.** Providers noted the need for high-quality professional development. Training should be free and online to ensure accessibility and incentivized using continuing education credits. Providers suggested incorporating ongoing professional development into providers’ yearly performance plans.
2. **Access to good**,** up-to-date resources and referrals.** Providers emphasized having current, readily available information on resources for patients with intersecting marginalized identities. They asserted that resources should be easily accessible and account for different presenting problems that clinics cannot address (e.g., human trafficking). Providers suggested that a responsible entity maintain the resources list.
3. **Up-to-date guidance.** Providers desired the latest and most accurate guidance available to provide LGBTQ+ affirming care from knowledgeable sources. This would increase clinician awareness of best practices when working with LGBTQ+ people, particularly practices that are “easy” to implement.

For staff, the top three recommendations in order of priority ratings were:

1. **Offer more safe spaces (e.g.**,** Safe Zones).** Safe spaces refer to an inclusive environment where a category of people will not be exposed to discrimination, criticism, harassment, or any other emotional or physical harm. Staff called for creating spaces in clinics, identified as Safe Zones. They noted that spaces should reflect an “ambient atmosphere” with LGBTQ+ safety signals (e.g., affirmative LGBTQ+ imagery) and staff trained to care for members of this population. They recommended spaces promote the promise that the clinic reflected a “judgment-free zone” free of heterosexist and cisgender bias.
2. **More transgender providers and services.** Staff called for increasing the availability of providers who identify as transgender, in addition to services staffed by people skilled in caring for transgender communities. Staff recommended increasing or redirecting funds to support this initiative.
3. **Educating employees in general.** Staff requested education for all employees because the topic of working with LGBTQ+ patients with intersecting marginalized identities is not discussed in their professional environments.

### Considerations for implementation

Providers and staff were asked to select topics from their NGT sessions that they wished to brainstorm ways to implement. Below, we present considerations for implementation based on their brainstorming.

#### Mandate ongoing professional development for providers and staff

In brainstorming about professional development, providers suggested methods to reach employees, including seminars during regular meetings and annual statewide conferences. They suggested tailoring training content for all staff, including resistant personnel. Moreover, training should be interactive (e.g., case vignettes, mentoring) and no longer than 4 h divided into 30–60-minute modules, with booster training as needed. Clinics could leverage and modify already-developed training materials. Providers should be aware of a state’s social climate and how it may harm LGBTQ+ people with intersecting marginalized identities. Participants stressed creating a “shame-free” environment for LGBTQ+ people of diverse cultural and social backgrounds. They emphasized regularly reviewing and adjusting professional development processes based on employee feedback and strong leadership buy-in to promote inclusivity. Another suggestion was changing licensing credentials to include training related to LGBTQ+ people of diverse cultural and social backgrounds.

#### Educating employees in general

Brainstorming among staff focused on educating employees by increasing engagement through in-person training, videos, and cartoons. Staff suggested methods to communicate information, including alerts in the electronic medical record, notices in communal areas, and dissemination during huddles. Staff advised against emails. Staff suggested organizational-wide training reinforced by supervisors. Training must consider constraints on employee time.

#### Safe spaces

Staff ideas for safe spaces involved formalizing education for all employees. Related ideas included recommending half-day training from the Safe Zone Project and integrating safe spaces into hiring processes and performance evaluations. In addition to employee-facing education on safe spaces, they reported the need to develop, post, and otherwise distribute patient-facing materials about safe spaces and how they are defined and incorporating visual cues signifying the clinic is a safe space (e.g., safety signals in entrances). They also recommended establishing upfront that their clinics do not discriminate against marginalized populations, including LGBTQ+ patients. Lastly, they mentioned creating and circulating similar materials advertising tangible assistance (e.g., food, hygiene/care packages) to patients to reduce structural barriers.

### Patient recommendations

The focus groups with LGBTQ+ patients offered an avenue to test the relevance and applicability of NGT-generated recommendations (Please see Appendix 2 for representative quotes). Patients endorsed recommendations similar to the ones described above. However, patients were concerned with highlighting heterogeneity within LGBTQ+ populations and considering intersectionality in caregiving contexts. They emphasized the importance of representation in primary care (e.g., hiring providers of color and who are transgender) and building capacity among personnel for critical reflection about their own backgrounds and the impact on care.

1. **Having providers who resemble the intersecting identities of the communities they serve.** Patients pondered whether providers could comprehend their lived experience, given that most in primary care settings did not reflect the demographic makeup of the larger community. A patient clarified, “One thing would be having providers that are not like majority white. Whenever I go to primary care…most of the people who are working there are white, and that’s obviously not really representative of [city] as a whole.” Similar to staff, patients called for hiring more transgender providers.
2. **Encouraging providers to develop capacity for critical reflection**,** including on their own background and its impact on care practices.** Even if providers do not share their intersecting marginalized identities or social realities, patients argued they must still consider these in delivering care. One focus group participant stated, “It’s okay if people don’t understand it 100%. There’s a lot of perspectives to have. Everyone is learning, but the only bridge for that gap is to have a genuine empathy for people who are different.”

Even when providers share the same identities as patients, commonalities should not be assumed. Patients advised providers to recognize and reflect on their positionalities, in addition to intracommunity differences between marginalized populations, and recommended education led by diverse trainers. To this point, one patient voiced, “Trans people aren’t one dimensional. There’s so many varieties and they’re always going to intersect with so many other things.”

## Discussion

This study sought to identify and prioritize recommendations for improving services for LGBTQ+ people from diverse racial, ethnic, cultural, and economic backgrounds by engaging providers, staff, and LGBTQ+ patients. At the provider level, recommendations emphasize professional development and access to resources. At the staff level, recommendations include offering more safe spaces, increasing providers’ preparedness to address transgender patient needs, and ensuring general education. Patients highlighted the importance of representation within clinics and the need for provider reflection on their backgrounds.

Provider and staff brainstorming sessions suggest developing and distributing educational materials, outreach, and ongoing training as potentially helpful implementation strategies for increasing equitable healthcare for LGBTQ+ people with intersecting marginalized identities [40, 41]. Participants identified several other implementation strategies, including altering incentive structures (integrating training and implementation of safe spaces into performance evaluations), changing credentialing or licensure standards, mandating change, promoting network weaving (e.g., creating a statewide conference), and changing physical structures and equipment (e.g., safety signals in entrances) [41]. 

One important note is that, despite the focal question, the highly structured NGT approach curtailed providers and staff from delving into discussing specific recommendations for LGBTQ+ people experiencing specific types of intersecting marginalized identities. However, all providers and staff worked in safety-net settings that predominantly cared for populations that are culturally diverse and of low income, which suggests that their recommendations are relevant across their clientele. Nevertheless, it still may be beneficial to integrate training related to intersectionality explicitly into provider and staff training programs and continuing education so that primary care professionals can enhance their understanding of intersectionality as a paradigm. Previous studies demonstrate that most students are unfamiliar with intersectionality theory and benefit from incorporating it into clinical learning [42, 43]. It is possible that including intersectionality in training and continuing education for providers as well as non-clinical staff may result in an increased understanding of intersectionality and the impact of interlocking oppression on LGBTQ+ patients and other groups with intersecting marginalized identities.

LGBTQ+ patients addressed intersectionality in focus group discussions by recognizing LGBTQ+ people are not one-dimensional. They provided suggestions to increase representation and, if this is not possible, to improve provider and staff understanding of LGBTQ+ people with intersecting marginalized identities. Clinics may consider re-evaluating their hiring practices to ensure they are inclusive to LGBTQ+ people who may wish to apply for jobs. Some practical considerations for inclusive hiring practices include using gender-inclusive language in job postings (e.g. they instead of he/she), including options for applicants to enter their affirming name and pronouns if they wish to do so, and specifically encouraging people with intersecting marginalized identities to apply [44, 45]. 

LGBTQ+ patients also suggested that providers engage in reflective practice and recognize intragroup differences within the LGBTQ+ community. One suggestion for doing this would be training in intersectionality, as mentioned above. It would be most beneficial for training to be conducted by people who themselves identify as LGBTQ+ people with marginalized intersecting identities, who can speak to their lived experience, and who receive payment for their labor. In addition to training in intersectionality, it may benefit providers and staff to engage in reflective practice – critically rethinking or debriefing on patient experiences and what behaviors staff and providers engaged in during their interactions with patients. This practice is especially beneficial when working with marginalized populations, such as LGBTQ+ patients with intersecting marginalized identities. Reflective practice is known to increase medical students’ critical thinking ability and engagement in learning [46]. However, providers and staff often are not allotted the time for reflective practice [47]. It is possible that setting aside time for providers and staff to engage in reflective practice may improve their ability to empathize with and understand LGBTQ+ patients with intersecting marginalized identities.

Outside of the U.S., barriers for LGBTQ+ patients with intersecting marginalized identities share some similarities including experiences of discrimination resulting in reluctance to access care [48]. Researchers in countries outside of the U.S (e.g., Australia, Canada) suggest addressing the needs of LGBTQ+ patients with intersecting marginalized identities through increasing connect to technology and providing patients with knowledge of the health care system and navigation support [49]. Similar to this study, some suggestions also include increasing representation of LGBTQ+ people with intersecting marginalized identities in health care as these individuals tend to be more aware of the intersecting needs of multiply marginalized populations [50]. It is likely the work done internationally may inform healthcare in the U.S. and vice versa.

These suggestions provide ways to consider patients’ unique needs based on identities (e.g., LGBTQ+ people of color’s experiences of racism, heterosexism, and cissexism). Although scholars are now providing thoughts on how to consider intersectionality in implementation efforts [8, 10], the application of intersectionality theory to implementation science is limited [51, 52]. The current study provides an example of how intersectionality can be considered in implementation efforts.

### Limitations

The study did not specifically recruit participants from diverse racial, ethnic, cultural, and economic backgrounds as it was part of a larger study; however, all of the LGBTQ+ patient participants held marginalized intersecting identities in some way, shape, or form (e.g., identifying as a sexual minority and disabled, identifying as a gender minority and not employed full time). Several of the LGBTQ+ patient participants discussed during focus groups that they would not be able to access care if not for the safety-net clinics serving their communities.

The limited number of NGT and focus group sessions and sample size curtails generalizability. Participants were invested in providing affirming care and may not reflect views of personnel with negative viewpoints. The recommendations derived from NGT may only be helpful to those who are neutral or positive towards LGBTQ+ people with intersecting marginalized identities. However, our focus group discussions with LGBTQ+ patients and findings from the larger study conducted at the clinics support these recommendations and make the case for investing in systemic change to enhance care for members of this population [27]. One additional limitation is that our NGT process only asked one question, “What are your recommendations to improve services for LGBTQ+ clients of diverse racial, ethnic, cultural and economic backgrounds?” It may be possible this question resulted in limited brainstorming and consideration of several different intersecting identities.

It may be beneficial to conduct future studies with more sizeable and diverse groups of LGBTQ+ patients, providers, and staff, which will allow for a deeper understanding of the primary care needs of LGBTQ+ patients with intersecting marginalized identities. Inclusion criteria for the current study included visiting a safety-net clinic for primary care within the past five years. It could be helpful to gather information from LGBTQ+ patients who have *not* visited such a clinic in the last five years to better understand their barriers to accessing primary care. Further, it may be useful to apply the NGT approach as a cost-effective and efficient means to develop more nuanced recommendations for specific subgroups within the LGBTQ+ community to respond to the unique needs of each group (e.g., Black, lesbian, transgender women may have different needs than South Asian, bisexual, cisgender women).

## Conclusions

All personnel highlighted continuing education and training as key to affirming care for LGBTQ+ people with intersecting marginalized identities. These recommendations were supported by the LGBTQ+ patients in focus groups, adding to the credibility of data collected through the NGT method. This method yielded insight into implementation strategies clinics can apply to create safe spaces and improve care. Further, we found that NGT is a feasible method to explore strategies for equitable implementation through targeted discussion of meeting LGBTQ+ patients with intersecting marginalized identities’ needs. Considering intersecting marginalized populations in implementation science research is helpful to ensure our implementation efforts are centered on promoting health equity.

## Data Availability

Data are available upon reasonable request to the Principal Investigator (CW).

## Appendix 1: Recommendations from staff and providers

### Staff session

#### Focal question

“What are your recommendations to improve services for LGBTQ clients of diverse racial, ethnic, cultural and economic backgrounds?”

**Table:**

Ideas from Frontline Staff
1. Better access to patient’s preferred name at first encounter (without having to look-up this information/extra effort)
2. Counseling (for both client and family) to provide emotional support and other assistance, such as case management and services for undocumented populations
3. **Educating employees in general because the topic is not talked about**
4. Awareness and attention to legal issues (ex. parents/decision-makers for non-married partners, legal name changes/gender changes)
5. Clinical education for providers regarding what LGBTQ clients may need
6. Post posters/pictures that are welcoming of LGBTQ patients and informational. Feature information that lets people know we are not judgmental. Consider distributing printed pamphlets/resources, and hanging flags and artwork showing alternative couples on wall)
7. Make available a phone number that community members can call to get needed assistance (ex. support for homelessness); resource directory
8. More classes/education for the public with information (regarding LGBTQ issues)
9. Patient assistance fund with set-aside [funds] for LGBTQ clients
10. Hold more workshops for underrepresented patients and patients who may not feel welcomed; ask LGBTQ patients what they need
11. **More transgender providers and services (both from the transgender community and people who are skilled in working with this community); more money to support these providers and services.**
12. **Offer more Safe Zones (places where patients feel safe and is ambient [atmosphere] with visuals and trained staff); promise patients that they are in a ‘judgement free zone’**
13. Access to prescription medications (getting insurance more on board [to reduce] patient expense; help patients with insurance coverage [to defray cost]
14. Access to safe emergency housing [that is] not just for ‘women’ (see Item #7)
15. Robust electronic medical system with [ready] access to preferred names, pronouns, and all other relevant patient information (across the board)
16. Supportive policies, such as patient nondiscrimination policy includes sexual orientation, and gender identity; such policies should be used and posted for patients; mechanisms should be in place at the clinic when policies are not followed
17. Have patient’s pronouns more [easily] accessible, and included in medical record; use correct pronouns [ties in with #1]
18. Have policy for making referrals [to LGBTQ affirmative services]
19. Educate people/other patients [tie in with pamphlets in waiting room]
20. Referral to LGBTQ-friendly services and supports; have directory [available to patients with phone numbers that they can call to obtain information related to LGBTQ topics, including legal issues such as name changes or parents who may be undocumented, medical decision making, and emotional support for LGBTQ people and their non-LGBTQ family member]
21. Hire LGBTQ staff (particularly for front office, including PSRs/MAs; staff [should be able to be] represented in clinic, out and talked about
22. Single use restrooms (not labeled male/female) in waiting and clinical areas
23. Education for staff and interpreters around queer words; interpretation challenges related to Spanish and Portuguese languages [noted and considered] evolving
24. Education to facilitate conversations with LGBTQ patients; [facilitate] open communication practices; [enable providers/staff to] follow patients’ lead
25. Change problematic questions on paperwork (i.e. Are you female or male?); “About Me” form example

### Provider session

#### Focal question

“What are your recommendations to improve services for LGBTQ clients of diverse racial, ethnic, cultural and economic backgrounds?”

**Table:**

Ideas from Direct Service Providers
1. How to work with other patients who might be prejudiced (and display prejudiced behavior in public spaces). Identify process to deal with these patients
2. Have team of participants from community (ex. community advisory group) come in/respond to complaints [rather than employees]; participants would function as ombudsmen and work with clinic to come up with response (ex. mediation). Characterized as new systemic solution…
3. Elevate safe, welcoming space, clinical practices [via auditing.] Existing regulatory agencies/entities [can] audit clinic to determine if it is safe and supports affirming clinical spaces/practices. Create internal audit tools to focus on best practices and quality improvement processes
4. Establish a specific clinic geared to LGBTQ for all age groups (to help address bullying issues, suicide, and more)
5. Establish a culture that “it is okay to ask” (for clinical staff/staff); [educate patients that they are empowered to] speak openly with their providers; culture [goes] both ways
6. Providers being mindful of LGBTQ needs [and ask patients about them]. Providers don’t ask about behavior/testing. However, they must know about LGBTQ needs [ex. HIV status sexual partners] to start conversations. This requires training; new territory for providers, so guidelines and professional development [may be necessary] [see Item #21]
7. Conduct outreach into community, meet persons [who are] shy of medical community where they’re at to build relationships
8. Make visibility-affirming materials available (e.g., rainbow flag or ‘subtle’ things) for patients to see when they first step [into the clinic]
9. Make intake paperwork friendly for everyone. Not usual demographics; patient can write what they need to write. Interpreters need to understand [the information that patients share so they can fit responses into boxes during charting]
10. **Mandate ongoing professional development for providers and staff. Professional development should be of high quality/online/include CMEs/built into performance evaluations. [Smaller clinics require professional development too and may be able to get it] free online. [See #26]**
11. Create a non-judgmental environment by educating staff about definitions (ex. (non-binary) and culture of LGBTQ so people understand. There are so many terms [used for] self-identifying; flip/cheat sheet may aid in understanding [terms and] needs
12. Allow for long enough visits (“double-slots”)
13. Establish stable set of designated providers who understand LGBTQ issues; assign patients to these providers for care [ex. ‘go to’ clinicians]; providers identify their specialties; break down walls and barriers
14. Have a consultant come to clinic to evaluate programming; [initiate this approach as] part of certification for clinics so people know [they are] safe place[s] to go
15. Set up gendered physical spaces [so] that all people can access [them] safely
16. Collect information on gender identity and sexual orientation info; make this information easily available to staff/clinicians. Do not ‘box in’ patients, but ask repeatedly for this information (ex. teen fluidity)
17. Capacity development across different models; we need capacity wherever people want to go (free-standing primary care clinic, patient-centered medical home, specialty clinic); do not send people away
18. **Access to good**,** up-to-date resources and referrals (ex. for Native American and transgender patients; include places both in and out of New Mexico that [providers] can access [for different needs**,** ex. substance abuse treatment and safety resources for persons who have been trafficked]; have a responsible entity maintain [list/directory] of resources/referrals.**
19. Have front staff and others asking patients, “What would you like to be called?” A 100-year old grandma might say “Mrs., no “Betty.”
20. [Provide education about] microaggressions (ex. what they are; [how they can be] unconscious/intrusive). Educate staff/asking questions that might [represent] a subtle slight or insult
21. [Increase] awareness of most common needs or issues among [LGBTQ people] [see Item #6]
22. **[Knowledgeable sources provide] updated guidance**,** best practices to clinicians that are easy to implement**
23. Ask right questions to individual persons [about] how they see themselves; allow [patients] to define labeling characteristics
24. Each clinic to have support groups for clients, families, partners/significant others, etc.
25. Learn how to take a very good sexual health history
26. Online CME [training] that is good and free [see Item #10]
27. Statistical knowledge of what the community faces (e.g. high rates of suicide) [see Items #21, #6]
28. Being able to have flexible assessment and treatment plans to [care for] the unique person with unique things about them; create a unique plan for those needs [versus cookie-cutter approach]
29. Put mechanisms in place to solicit anonymous patients reports (ex. experiences/care incidents in clinic); [a lot of time patients don’t report] link [this item to] ombudsman
30. [Establish] safe environment (ex. physical, emotional, overall environmental [that includes protocols for] how to deal with people in lobby)

## Appendix 2: Representative quotes from LGBTQ+ patient focus groups

**Table:**

Theme	Quotes
Recognize heterogeneity within LGBTQ+ populations	“We’re in the state of 135 Native American tribes […] I’m just fortunate enough to be a part of the Navajo tribe although there’s all this struggle of who I am, even my age, […] and being homeless” (FG 01)
Representation in primary care (e.g., hiring more providers with intersecting marginalized identities)	“I don’t recall ever seeing people of—I don’t recall ever seeing minorities in my clinic. Maybe I just don’t remember it, but my clinic seems very much a white person clinic.” (FG 01)
Encourage providers to develop capacity for critical reflection	“It’s almost like you know people just need to fundamentally understand that oppression is a real thing, feel it for themselves, have some empathy and then go into every situation like that.” (FG 02)
Education led by diverse trainers	“Hire trans people. Hire them, bring them in, that solves a lot of problems. Then they have money and opportunity but also, I don’t know I think I would just be afraid too that like situations where you’re interacting with them one-on-one is not taught by a trans person that always can get dicey” (FG 02)

## Abbreviations

FQHC: Federally Qualified Healthcare Center
LGBTQ+: Lesbian, gay, bisexual, transgender, queer+
NGT: Nominal group technique
SAB: Scientific advisory board
US: United States
